# Comparative Experimental Study of a High-Temperature Raman-Based Distributed Optical Fiber Sensor with Different Special Fibers

**DOI:** 10.3390/s19030574

**Published:** 2019-01-30

**Authors:** Ismail Laarossi, María Ángeles Quintela-Incera, José Miguel López-Higuera

**Affiliations:** 1 Photonics Engineering Group, University of Cantabria, 39005 Santander, Spain; miguel.lopezhiguera@unican.es; 2 CIBER-bbn, Instituto de Salud Carlos III, 28029 Madrid, Spain; 3 Instituto de Investigación Sanitaria Valdecilla (IDIVAL), 39011 Santander, Spain

**Keywords:** distributed sensor, Raman DTS, Optical-Time-Domain-Reflectometry, temperature sensor, fiber optics sensors, high temperature

## Abstract

An experimental study of a high temperature distributed optical fiber sensor based on Raman Optical-Time-Domain-Reflectometry (ROTDR) (up to 450 °C) and optical fibers with different coatings (polyimide/carbon, copper, aluminum and gold) is presented. Analysis of the distributed temperature sensor (DTS) measurements determined the most appropriate optical fiber to be used in high temperature industrial environment over long periods of time. To demonstrate the feasibility of this DTS for an industrial application, an optical cable was designed with the appropriate optical fiber and it was hermetically sealed to provide the required mechanical resistance and isolate the fiber from environmental degradations. This cable was used to measure temperature up to 360 °C of an industrial furnace during 7 days.

## 1. Introduction

Distributed temperature sensors based on the spontaneous Raman effect have become widely used in oil and gas sectors in applications such as leak detection in oil wells and thermal control of power lines [1,2,3,4]. These sensors offer many advantages in comparison to standard discrete temperature sensors [5,6] including inherent immunity to electromagnetic fields and allowing remote, long distance temperature measurement in dangerous, radioactive and toxic environments [7,8]. In recent years, this type of system has been an intense area of research with the aim of enhancing the systems’ performance, whether by improving the spatial and temperature resolution [9,10,11,12] or maximizing the detection length [13].

Despite the many advantages of Raman DTS sensors, they are limited to measurements in environments that do not exceed 300 °C. This limitation has created a demand for sensors able to monitor temperatures with precision and for long periods of time in the hostile environments (>300 °C) common in oil or gas industrial sectors.

To the best of our knowledge, distributed temperature sensors have not been proved to fulfill this demand for real industrial applications. Prototypes have been developed and presented in scientific papers, but testing and application of these sensors has been limited to highly controlled laboratory scenarios. In [14], DTS measurements in geothermal wells were reported up to temperatures of only approximately 300 °C during continuous operation. In [15], the authors presented 1200 °C distributed sensing based on a pure-silica photonics crystal fiber, but the fiber was tested in a highly controlled laboratory setting. In [16], a distributed temperature measurement up to 600 °C with a gold-coated fiber was carried out. However, as we will discuss here, the drawbacks of gold-coated fibers make them unsuitable for use in most industrial applications.

The optical fiber as transducer is the limiting factor in the development of robust and practical distributed high temperature sensors in extreme industrial environments. Optical fibers, under laboratory conditions in the absence of stress, curvature and movement, can withstand temperatures above 600 °C even without protective coatings. However, in industrial applications the mechanical consistency of optical fibers can be compromised, which can cause sensor measurement errors as well as precipitate the breakage of the fiber. For these reasons, fibers for use in an industrial setting require a coating that withstands extended use under extreme conditions and temperatures.

The following types of protective coatings are currently available on the market: polyimide and metal (aluminum, copper and gold) [17,18]. Although each type of coating is designed for industrial use at high temperatures, their advantages and disadvantages should be considered.

Polyimide coating is very common, but it is limited to applications where temperature does not exceed 300 °C, according to the technical specifications of the manufacturers of these fibers. Additionally, polyimide-coated fibers are not impermeable and can undergo core hydrogen penetration, which increases attenuation in certain wavelengths of the infrared spectrum and reduces fatigue stress resistance [19]. To prevent the problems associated with hydrogen penetration a coating of carbon is applied to the fiber during manufacturing to block the entry of water and slow down the diffusion of hydrogen. The coating is thin and does not cause any optical loss, and it is functional even for long lengths of fiber. However, carbon coating of polyimide optical fiber coatings is not completely hermetic and its effectiveness as a hydrogen barrier decreases as temperature increases.

Metal coatings also create a hermetic layer that protects the fiber optic core from hydrogen penetration. The advantages of metal coatings, in comparison to polyimide, include their resistance to higher temperature environments (>350 °C), nuclear radiation (polymer coating decays under radiation) and certain chemicals [20]. Metal coatings also work theoretically in environmental temperatures close to the melting point of the specific metal; however, despite these advantages, metal coatings, whether they be aluminum, copper, or gold, can react unpredictably with silica at high temperatures.

For example, aluminum has a melting point of 660 °C in its pure state, but aluminum coated fibers can only be used in long-term applications at temperatures < 400 °C due to the reaction that occurs between aluminum and silica, which causes the fiber strength to degrade quickly [21].

Copper does not react with silica at high temperatures and it has a higher melting point than aluminum (approximately 1000 °C), which makes it a better option for high temperature applications [22]. However, the oxidation process on the copper surface in the presence of oxygen at high temperatures causes a deterioration of the mechanical properties of copper coated fibers [23].

Gold-coated optical fibers are another solution that has been recently proposed [16]. These fibers have a high attenuation at low temperatures; however, as temperature increases, a decrease in attenuation is observed [24]. These dynamic and non-uniform variations in the attenuation profile are generally attributed to micro-bending and to the major discrepancy of the coefficients of thermal expansion between the gold coating and glass fiber. This behavior implies that conventional calibration techniques can not be employed, which is a disadvantage. In comparison with other coating materials, gold coated optical fibers are also more costly and fragile and require careful handling, two factors which can increase the final price of the system.

This paper presents an experimental study of a high temperature distributed sensor system using a Raman-Optical-Time-Domain-Reflectometry (ROTDR) (up to 450 °C) with different optical fiber coatings (polyimide/carbon, copper, aluminum and gold). The objective is to determine which fiber performs with higher precision and reliability in harsh environments over long periods of time. DTS measurements with each fiber were made at different temperature cycles. The results obtained will allow us to determine the behavior of each fiber as temperature increases and therefore calculate their influence on the accuracy of the measurements in a Raman DTS system at high temperatures.

## 2. Operating Principles

**Figure 1:**
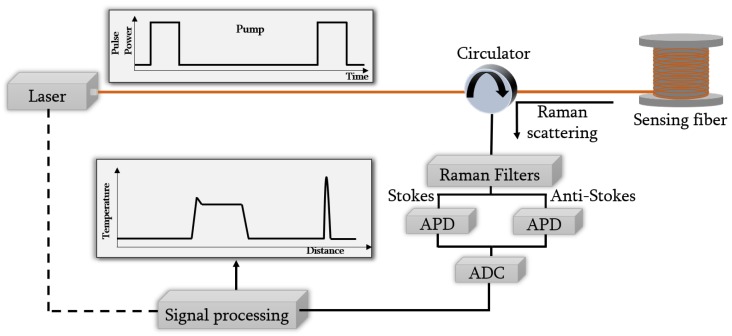
Schematic diagram of Raman distributed temperature sensor system based on optical time domain reflectometry (OTDR) technique.

In Figure 1, a typical schematic diagram of Raman DTS system based on OTDR technique is shown.

Most Raman DTS systems are based on the principle of optical time domain reflectometry (OTDR), in which a pulsed light source is used to provide probe pulses at the pump wavelength ($\lambda_{p}$). The probe pulses are coupled into one end of the fiber under test through a circulator [3]. Along their propagation, the pulses interact with the fiber core molecules and therefore, Raman scattering is generated. A small portion of this scattered light, containing information about loss and temperature along the entire fiber, is guided back to the launching end. The position of the scattering in the fiber can be calculated by the measurement of the arrival time of the returning light and by the speed of light in the fiber. Using a suitable optical filter, the Raman backscattered light is separated from the other backscattered signals and, additionally, it is also separated into its both components.

The Raman backscattered signals recovered are characterized by their low power values, about 30 dB weaker than Rayleigh backscattering light [25]. Hence, a high-sensitivity low-noise avalanche photodiodes (APD) are needed to detect the filtered optical signals. Once these signals are detected, they are sampled using a high-resolution analog-to-digital conversion (ADC) system.

In the Raman scattering process, the pumping light interacts with the molecular vibrations, producing an energy exchange between the incident photons and the vibration states of the molecule. Therefore, the characteristic vibrational frequencies of the molecules determine the Raman frequency shift. In silica, its value is approximately Δ*f* = 13.2 THz (50 and 100 nm for a pumping wavelength of 1064 and 1550 nm, respectively) [26].

The process in which the emitted photon has a lower energy than the incident photon, and therefore, its frequency is also reduced, it is named Stokes (S) Raman scattering. In the opposite case, when the emitted photon has a higher energy than the incident photon, gaining energy in the process, the process is known as anti-Stokes (AS) Raman scattering [27].

The intensity of the anti-Stokes component is strongly dependent on temperature [28], but as mentioned above, depends also on other parameters such as the attenuation at AS wavelength, local losses in the fiber, etc. To overcome this problem, the ratio of anti-Stokes intensity to Rayleigh or Stokes intensities is normally used [29].

In the case of using the two Raman backscattered signals, the ratio between the power of both components ($P_{AS}\left(z,t\right)$ and $P_{S}\left(z,t\right)$) can be expressed as [30]:

(1) $$R\left(z,t\right)=\frac{P_{AS}\left(z,t\right)}{P_{S}\left(z,t\right)}={\left(\frac{\lambda_{S}}{\lambda_{AS}}\right)}^{4}e^{\frac{-h\Delta v}{KT}}e^{-\int_{0}^{z}\Delta \alpha \left(z^{\prime }\right)dz^{\prime }}$$

where *T* is the absolute temperature, *z* is the distance along the fiber and $\lambda_{S}$ and $\lambda_{AS}$ are the Stokes and anti-Stokes wavelengths respectively. Δα = $\alpha_{AS}-\alpha_{S}$ is the differential attenuation, considering that $\alpha_{S}$ and $\alpha_{AS}$ are the fiber attenuation coefficients at $\lambda_{S}$ and $\lambda_{AS}$ respectively; Δ*v* is the frequency separation between AS and pump signal, h is the Planck constant and k is the Boltzmann constant.

From the ratio equation of the anti-Stokes and Stokes signals, the temperature profile along the fiber can be calculated according to the equation [31]:

(2) $$T\left(z,t\right)=\frac{\gamma }{ln\left[\frac{P_{S}\left(z,t\right)}{P_{AS}\left(z,t\right)}\right]+C\left(t\right)-\int_{0}^{z}\Delta \alpha \left(z^{\prime }\right)dz^{\prime }},$$

where γ is defined as hΔα/k and *C*(*t*) is dependent on the instrument temperature and it is not a function of position along the cable [31]. Therefore, we consider it invariant over time given that the DTS system temperature does not change. Finally, the term $\int_{0}^{z}\Delta \alpha \left(z^{\prime }\right)dz^{\prime }$ stands for the accumulative differential attenuation of the Stokes and anti-Stokes backscatter light along the fiber.

For a single-ended Raman DTS measurement based in OTDR, a calibration must be carried out to obtain the parameters: Δα, *C* and γ [32], before beginning the measurements. This is normally achieved maintaining a section at known, uniform and stable temperature. The value of the differential attenuation in this section can be calculated as the slope of the line that adjusts $\ln\left[R(z,T)\right]$ with *z* (considering that the optical fiber maintains its attenuation spectrum over time and therefore, Δα remains constant), as can be deduced from the following equation:

(3) $$ln\left[R\left(z,t\right)\right]=\frac{-h\Delta v}{KT}+ln\left[{\left(\frac{\lambda_{S}}{\lambda_{AS}}\right)}^{4}\right]-\Delta \alpha z$$

γ can be fixed to its theoretical value γ = 490 K [31] for a laser emitting in 1064 nm and finally, the constant *C* can be estimated from the Equation (2) in the reference section.

## 3. Experiments

**Figure 2:**
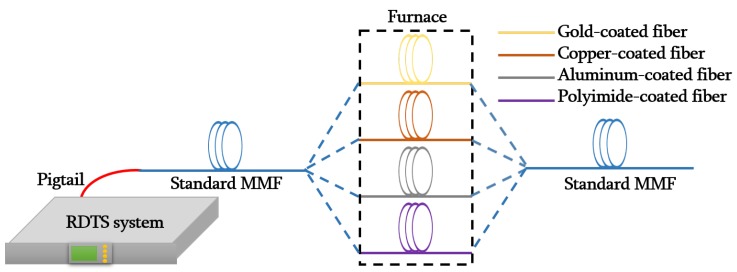
Schematic diagram of the set-up used in the experimental tests: Raman-based Distributed Temperature Sensor instrument (RDTS). Sensing fiber employed with standard multimode graded index fiber (blue) and special coated fibers: gold-coated fiber of 76 m length (yellow), copper-coated fiber of 40 m length (brown), aluminum-coated fiber of 64 m length (grey) and polyimide-coated fiber of 14 m length (purple).

The experimental set-up used to carry out the measurements is shown in Figure 2. A Distributed Temperature Sensor (DTS) interrogator based on Raman Optical-Time-Domain-Reflectometry (ROTDR) was used to accomplish the measurements. The Raman DTS source is provided by a laser operating at 1064 nm with an average output power of 17 mW.

The fibers under test (FUT) used in the set-up are formed by a standard graded-index multimode fiber (MMF-50/125 µm) and by graded index multimode pure silica core fibers with different coatings: carbon and polyimide (Fibercore GIMMSC(50/125)CP), copper (IVG Fiber Cu50-125), aluminum (Fibercore GIMM(50/125)AL) and gold (FiberGuide AFS50/125/155G). A section of the polyimide and metal coated fibers were inserted into a high temperature controlled chamber. Table 1 lists the characteristics of the different fibers that have been tested.

To carry out the measurements with each fiber, a multimode pigtail has been used to connect the RDTS system with the different standard multimode fibers, which are used to facilitate access to the special coated fibers used in this work.

Different thermal cycles that simulate conditions similar to industrial environments have been programmed. This experimental thermal study allows for determination of the optical and mechanical behavior of the special fibers when they are subjected to temperature changes. In the first cycle of the first series of measurements, the furnace temperature was increased from room temperature (20 °C approximately) until the maximum testing temperature was reached (see Table 1) with steps of 25 °C, except for the gold-coated fiber where the maximum temperature was 450 °C. After changing the temperature and before taking the measurements, 20 min were needed until the temperature was stable in the furnace chamber.

**Figure 3:**
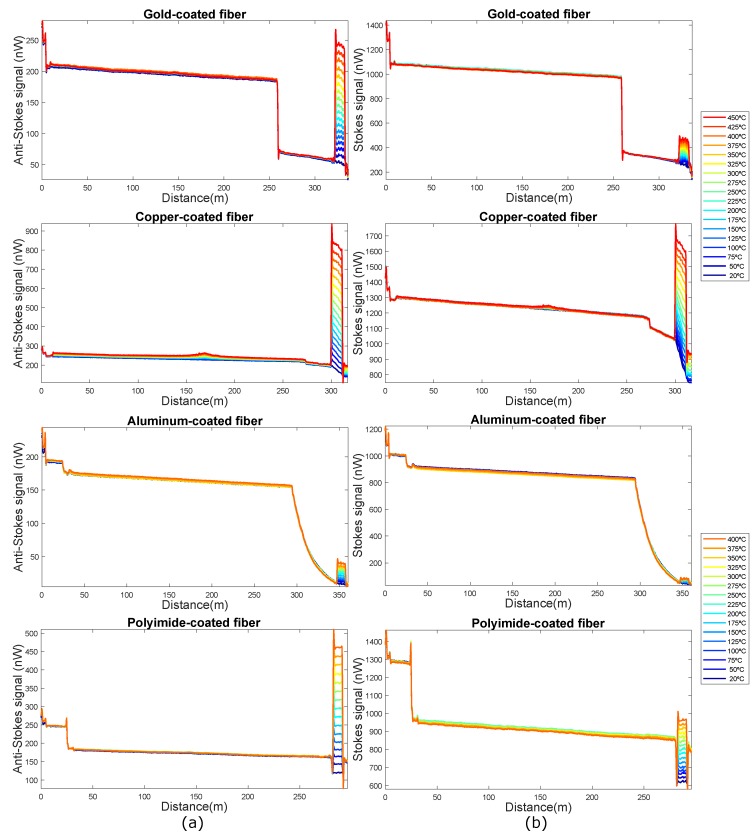
(**a**) Anti-Stokes and (**b**) Stokes signals obtained for gold-coated, copper-coated, aluminum-coated and polyimide/carbon-coated fibers (from top to bottom respectively) in the entire fibers under test (FUT).

The anti-Stokes and Stokes signal traces have been obtained with the RDTS instrument (at the $\lambda_{AS}$ = 1014 nm and $\lambda_{S}$ = 1114 nm wavelengths) for the four optical fibers with a spatial resolution of 0.5 m (to achieve this resolution, pulses with 5 ns duration have been employed). The sampling resolution value has been chosen to be half the spatial resolution (0.25 m). The results for the entire FUT are shown in Figure 3.

**Figure 4:**
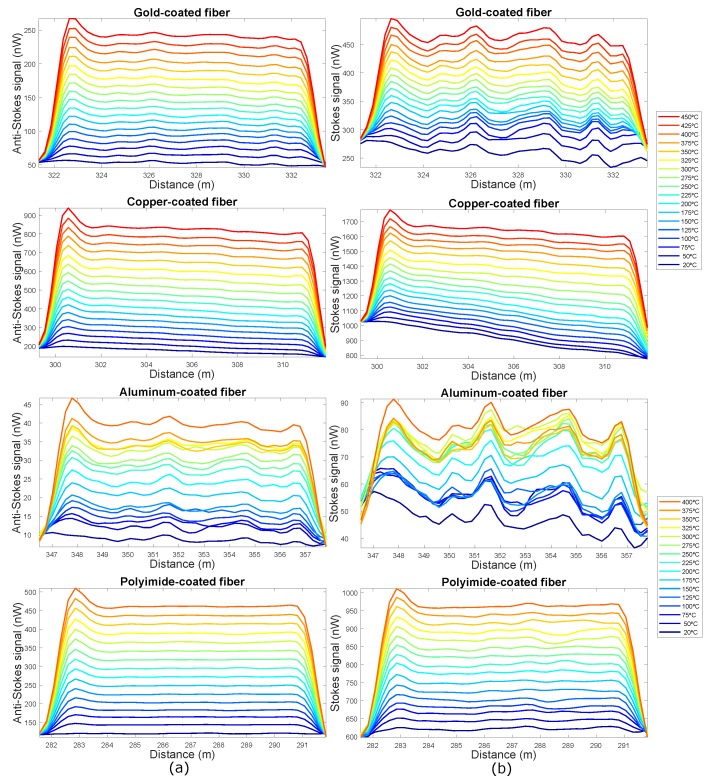
(**a**) Anti-Stokes and (**b**) Stokes signals obtained for gold-coated, copper-coated, aluminum-coated and polyimide/carbon-coated fibers (from top to bottom respectively) in the hot-spot zone.

For all fibers, the anti-Stokes signal is weaker than the Stokes signal, as expected. However, in the section where the fibers are at higher temperatures, the anti-Stokes signal shows a greater increase with temperature than the Stokes signal. Therefore, this distributed measurement technique based on Raman is very sensitive to temperature. In addition, it can also be observed that in the section of the trace corresponding to the standard MMF fiber that is outside the oven at room temperature, both anti-Stokes and Stokes signals have higher power levels than in the section of the traces corresponding to the fibers with special coating. This fact can be clearly noticed as an abrupt decay in the power of the signals shown in Figure 3. This is due, on the one hand, to the backscattered signals generated in pure fused silica fibers are less than the generated in standard MMF and on the other hand, to the local losses produced in the fusion of the standard MMF fiber and the special coated fibers. Furthermore, in the aluminum-coated fiber, the exponential decrease can be noted despite the small length of the fiber, because of the high attenuation value of this type of fibers. If we zoom into the hot-spot zone, we can see in more detail the evolution of the Stokes and Anti-Stokes signals with temperature for the different fibers (Figure 4). This allows us to obtain information about the behavior of these fibers with temperature.

It can be observed that at the start of the section where the temperature changes, the trace presents the form of “S”, due to the chromatic dispersion effect [31].

The anti-Stokes and Stokes signals traces of the aluminum and gold-coated fibers are noisier than the traces of the polyimide and copper-coated fibers. The reason for this excess noise is the macro-bending that these fibers have supported when they were introduced into the oven. The four fibers were placed in the oven in the same way, but in view of these results the deployment and curvatures of the aluminum and gold coated fibers influence the results. This means that these fibers may not be suitable for use in industrial environments, where most of the time the deployment or handling of these fibers cannot be controlled.

The optical attenuation of the polyimide/carbon and metal coated fiber depends on temperature: for low temperature they present higher attenuations [24]. In the graph associated with the copper-coated fiber of Figure 4, it can be observed that the Stokes and Anti-Stokes signals at ambient temperature (20 °C) present a negative slope due to attenuation. When temperature increases this slope decreases, which means that attenuation decreases. For the polyimide fiber, this variation can not be appreciated from the traces obtained with this interrogator given that its attenuation is lower and there are few meters of fiber, thus the resolution of the system does not allow to obtain it with precision. This variation of the attenuation with temperature is not appreciated in the anti-Stokes and Stokes signal traces of the aluminum and gold coated fibers, because they are non-uniformity traces, which is due to the macrobends of the fiber when it is introduced into the oven.

**Figure 5:**
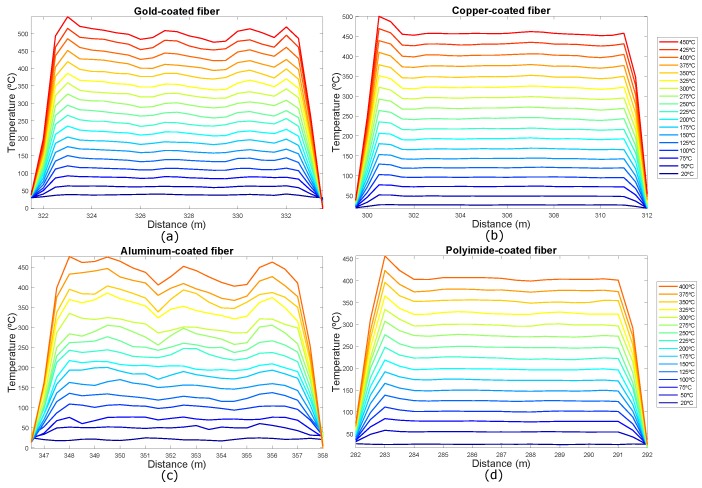
Temperature profile for (**a**) gold-coated; (**b**) copper-coated; (**c**) aluminum-coated and (**d**) polyimide/carbon-coated fibers in the hot-spot zone.

From these recovered Raman backscattered signals in the FUTs, the temperature profiles of the optical fibers have been estimated. Figure 5 shows the estimated temperature in the hot-spot section for the four FTUs.

**Figure 6:**
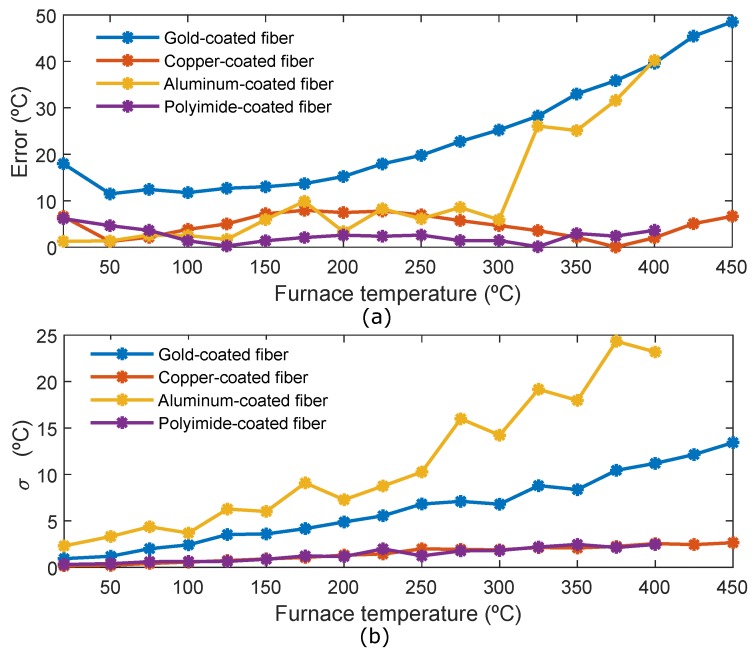
(**a**) Mean error of the hot-spot section for the four fibers; (**b**) Variance (σ) of the hot-spot section for the four fibers.

Figure 6a shows the mean error of the hot-spot section for the four fibers. The error has been calculated as the absolute value of the difference between the real oven temperature and the average temperature of the hot-spot section. The polyimide-coated fiber is the fiber that presents the minimum error, remaining constant with temperature. The highest error obtained is 6 °C at room temperature (probably because the room temperature has been considered 20 °C to calculate the error in all the fibers, however small changes could have been produced in this value when the measurements with polyimide fiber were carried out). Copper coated fiber also has a small and practically constant error with temperature, the biggest error is 7.9 °C for temperature = 175 °C. In the aluminum-coated fiber the error triggers from 300 °C, and, its error is almost 40 °C at 400 °C. The gold-coated fiber is the one with the biggest error, which increases with the temperature.

This greater error in the measurements with the aluminum and gold coated fibers is due to the non-uniform change of the losses with temperature in the wavelength of S and AS signals, and this has an impact on the calculation of the Δα parameter, which is very important in performing a correct calibration. The explanation of this behavior is found in the loss microbendings due to the difference between the coefficients of linear thermal expansion of optical fiber and metal coating.

To reduce this error, a dynamic calibration should be performed to compensate the variations in the value of the accumulative differential attenuation. In [16] a possible method to carry out the dynamic calibration process is explained in detail. The proposed solution here consists of dividing the channel into several sections, which correspond to different types of fibers or sections at different temperatures. Once it is determined that the temperature is stable along the entire channel, the accumulative differential attenuation values are calculated in each fiber section for each temperature. It is important to note that in a dynamic calibration, for each hot spot section, the system must be re-calibrated after each temperature change. This means that these metal-coated fibers can be used exclusively in industrial applications where the temperature to be measured is known and the thermal changes are slow and stable.

In Figure 6b, the variance of the hot-spot section for the four fibers is shown. The variance of the polyimide and copper coated fibers is very small and uniform with the temperature. However, the variance of the gold and especially aluminum coated fibers increases with temperature, which has a direct relationship with the non-uniformity of the Stokes and Anti-Stokes signal traces (Figure 4). In view of the results of Figure 4 and Figure 6b, several conclusions can be drawn. On the one hand, the deployment and curvatures of the aluminum and gold coated fibers influence the results of the measurements. This means that these fibers may not be suitable for use in industrial environments, where most of the time the placement or handling of these fibers can not be controlled. On the other hand, in an industrial application where the exact temperature value of a specific area of small length is needed, the high value of the variance for the aluminum and gold-coated fibers limits the use of these fibers in this application.

**Figure 7:**
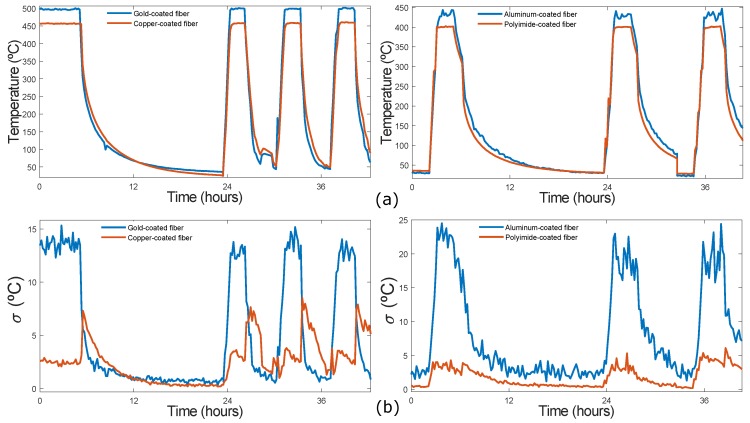
(**a**) The average temperature of the hot-spot section for the four fibers; (**b**) variance (σ) of the hot-spot section for the four fibers.

In the second series of measurements, the furnace temperature was changed between room and maximum testing temperatures (see Table 1). Temperature measurements of gold and copper-coated fibers, on the one hand and aluminum and polyimide-coated fibers on the other hand, were performed at the same time. The average temperature of the hot-spot section for the four fibers is shown in Figure 7a. As previously explained, the gold and aluminum-coated fibers present higher errors (50 °C and 40 °C, respectively) at maximum test temperatures. Moreover, in the case of the aluminum-coated fiber the average temperature of the hot-spot section is not constant when the furnace temperature is stable. In contrast, both copper and polyimide-coated fibers exhibit lower and constant errors (7 °C and 3 °C respectively at maximum test temperature) during these cycles.

The variance of the hot-spot section for the four fibers is shown in Figure 7b. It can be observed, as in previous results, that the variance associated with both gold and aluminum-coated fibers is higher (reaching 25 °C approximately in the aluminum-coated fiber). In the case of the copper-coated fiber, the variance is lower, except when the temperature is ramped down to room temperature. This is probably because the fiber inside the furnace is not kept constant. Finally, the polyimide-coated fiber exhibits a low and constant variance in all cycles.

Once the experimental study has been carried out to determine the advantages and disadvantages of the different fibers to measure high temperatures with a Raman DTS system, we can conclude that copper and polyimide-coated fibers are the best option. Copper-coated fibers may be a very suitable option, but present the problem of oxidation at high temperatures. While this problem is not solved, polyimide-coated fibers will be the best option as long as adequate protection is chosen to give them the required mechanical consistency at high temperatures.

To demonstrate the viability and reliability of this DTS System with a polyimide-coated fiber for an industrial application, 30 m of the FUT, which was protected by a loose shield hermetically sealed to offer the required mechanical resistance and isolate the fiber from environmental degradations, was used to measure the temperature. The last 22 m of this fiber was inserted into an industrial furnace for 7 days. Two temperature cycles between room temperature and 360 °C were carried out, as shown in Table 2.

**Figure 8:**
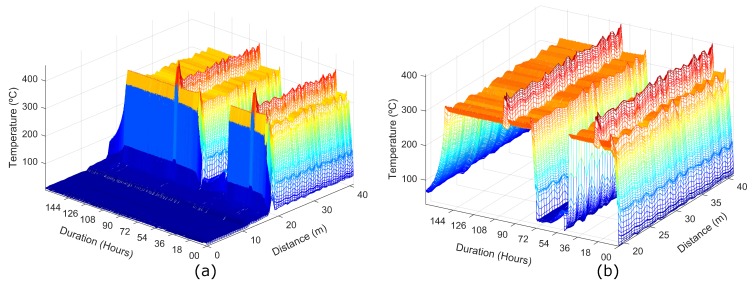
Temperature distributed measurements; (**a**) Entire FUT; (**b**) Hot-spot zone.

Figure 8a shows the results of the temperature distributed measurements performed using a spatial resolution of 0.5 m. The measurement time of each trace and the time between two consecutive measurements were 60 s and 5 min, respectively. For a clear observation of the two temperature cycles applied to the fiber, Figure 8b presents only the hot-spot zone.

**Figure 9:**
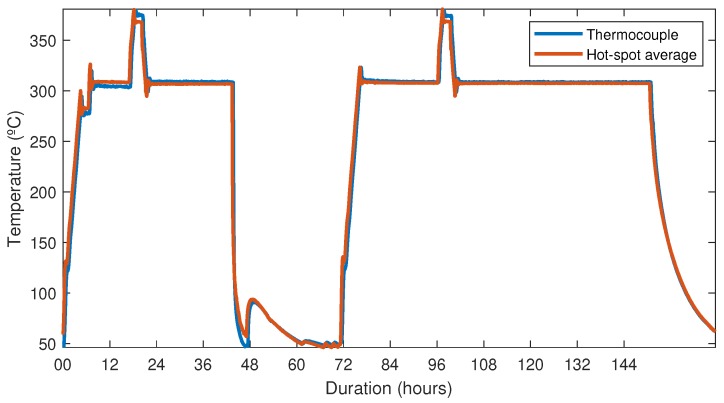
Comparison between the values recorded by the thermocouple and the average of the hot-spot section.

With the purpose of monitoring the temperature at the top of the cable inside the furnace chamber, a calibrated thermocouple was placed on the fiber cable surface. A comparison between the values recorded by the thermocouple and the average of the hot-spot section is shown in Figure 9. As can be appreciated, both values show the same results, the only difference is the desynchronization between the data captured by the thermocouple and the RTDS instrument. The maximum error committed in determining the temperature when the furnace was stable at 300 °C was approximately 4 °C.

After finishing the measurements, the state of both the cable and the fiber was checked and it was verified that both of them have maintained their initial physical and mechanical properties. Therefore, the proposed approach in this work meets the objectives for which it was designed, and it can be an attractive solution for applications where high temperature monitoring is required, such as in oil and gas sectors, metallurgy and in the nuclear industry. Furthermore, the carbon layer in the proposed fiber provides a hermetic barrier to water and hydrogen, which makes it a suitable fiber cable in hydrogen-rich environments, such as geothermal wells.

## 4. Conclusions

This work arises from the need to measure high temperatures (above 300 °C) for long periods of time in industrial environments. Conventional fibers can not be used because the acrylate coating disappears at those temperatures and the fiber becomes extremely brittle. This implies that there is a need to use special coated fibers to give them the protection required to maintain their physical and optical properties. A high-temperature distributed sensing system based on a ROTDR implementation and four types of fibers: multimode polyimide/carbon-coated, multimode copper-coated, multimode aluminum-coated and multimode gold-coated fibers has been presented in this paper. In addition, a comparison between these fibers has allowed us to determine which is the most suitable coating to measure high temperatures in industrial environments over long periods of time. In view of the experimental results, among these four fibers, the best options are polyimide/carbon and copper-coated fibers. However, copper-coated fibers have the great disadvantage of oxidation at high temperatures. This DTS system with 30 m of the polyimide/carbon-coated fiber, which was protected by a loose shield hermetically sealed to offer the required mechanical resistance and isolate the fiber from environmental degradations, was used in an industrial application. The temperature was measured between room temperature and 360 °C for 7 consecutive days.

## Figures and Tables

**Table 1:** List of tested fibers with respective coatings. Maximum operating temperature (manufacturer specification). Length of the fiber inserted into a high temperature controlled chamber.

Sample	Coating (Diameter (µm))	Max. Temperature (°C)	Length (m)
1	Gold (155)	700	10.5
2	Copper (165)	450	11.5
3	Aluminum (175)	400	10.5
4	Carbon/Polyimide (155)	400	10

**Table 2:** Temperature cycles between room temperature and 360 °C.

	Cycle I				Cycle II				
	I	II	III	IV	I	II	III	IV	V
**Temperature (°C)**	Room→300	300	360	300	300→Room→300	300	360	300	300→Room
**Duration (hours)**	8	9	5	21	34	18	5	50	18
